# Poster Session II – Poster of Distinction II - A208 CONNECTING ADVERSE FOOD REACTION TO GUT INFLAMMATION: DISRUPTION OF MICROBIAL METABOLISM BY GUT INFLAMMATION DRIVES ADVERSE FOOD REACTIONS

**DOI:** 10.1093/jcag/gwaf042.208

**Published:** 2026-02-13

**Authors:** B Barbosa da Luz, L Rondeau, P Muppidi, F A Vicentini, G De Palma, P Bercik, A Caminero

**Affiliations:** McMaster University, Hamilton, ON, Canada; Medicine, McMaster University, Hamilton, ON, Canada; McMaster University, Hamilton, ON, Canada; McMaster University, Hamilton, ON, Canada; McMaster University, Hamilton, ON, Canada; McMaster University, Hamilton, ON, Canada; McMaster University, Hamilton, ON, Canada

## Abstract

**Background:**

Patients with inflammatory bowel disease (IBD) experience diverse food-related adverse reactions. However, mechanisms underlying food intolerances in IBD remain unclear. We hypothesized that inflammation-driven microbiota alterations contribute to the development of adverse food reactions.

**Aims:**

Determine the role of inflammation and microbial metabolism in adverse food reactions.

**Methods:**

Specific pathogen-free (SPF) C57BL/6 mice with DSS or TNBS-induced colitis were sensitized to dairy protein using cholera toxin during the final phase of inflammation, and placed on either a dairy-containing or control diet for 7 days. Cromolyn sodium was provided daily during the diet intervention in a subset of mice to study the role of mast cells. To assess the microbiota’s role in inflammation-induced food sensitization, germ-free mice were colonized with SPF or DSS-induced dysbiotic microbiota (post-DSS), and sensitized to dairy proteins. To test if depleted bacteria after inflammation reduce adverse food reactions, SPF C57BL/6 mice were sensitized with dairy after inflammation, and supplemented with bacteria degrading dairy or sham during the dietary intervention. Markers of intestinal inflammation (histological damage, CD3^+^ cell infiltration) and pro-inflammatory gene expression (NanoString) and food sensitization (IgE and mast cell responses-mMCPT-1), as well as visceral hypersensitivity and intestinal microbiota were analysed.

**Results:**

Intestinal inflammation, whether induced by DSS or TNBS, promotes sensitization to dairy proteins by impairing microbial digestion of dairy proteins, recruiting mast cells to the colon, and enhancing mucosal production of IgE. Upon re-exposure to these foods, sensitized mice experienced immune infiltration, visceral hypersensitivity, increased apoptotic cells, and low-grade inflammation. This response is mediated by mast cell–dependent mechanisms, as treatment with cromolyn sodium reverses the phenotype. In gnotobiotic models, post-DSS microbiota promoted food sensitization, suggesting that inflammation-driven loss of colonic bacteria involved in dairy digestion contributes to this process. Finally, supplementation with dairy-degrading bacteria attenuated food-related immune responses in our model.

**Conclusions:**

Gut inflammation impairs microbial metabolism, promoting sensitization to food proteins via mast-cell mediated mechanisms, which may underlie adverse food reactions in some IBD patients. Our results suggest that modulating the intestinal microbiota, for instance through probiotics capable of degrading dairy, could help reduce adverse food reactions in IBD patients.

**Funding Agencies:**

CCCTRIANGLE

